# MicroRNA-449c-5p inhibits osteogenic differentiation of human VICs through Smad4-mediated pathway

**DOI:** 10.1038/s41598-017-09390-z

**Published:** 2017-08-18

**Authors:** Rongjian Xu, Min Zhao, Yun Yang, Zhuo Huang, Chunying Shi, Xianglin Hou, Yannan Zhao, Bing Chen, Zhifeng Xiao, Jianzhou Liu, Qi Miao, Jianwu Dai

**Affiliations:** 1 Department of Thoracic Surgery, The Affiliated Hospital of Qingdao University, Qingdao, China; 2 Center of Laboratory Medicine, Qilu Hospital of Shandong University (Qingdao), Qingdao, 266035 China; 3 State Key Laboratory of Molecular Developmental Biology, Institute of Genetics and Developmental Biology, Chinese Academy of Sciences, Beijing, China; 4 Graduate School, Chinese Academy of Sciences, Beijing, China; 5 Department of Cardiac Surgery, Peking Union Medical College Hospital, Peking Union Medical College, Chinese Academy of Medical Sciences, Beijing, China; 6 Institute for Translational Medicine, College of Medicine, Qingdao University, Qingdao, China; 7 Institute of Combined Injury, State Key Laboratory of Trauma, Burns and Combined Injury, College of Preventive Medicine, Third Military Medical University, 30, Gaotanyan Road, Chongqing, China

## Abstract

Calcific aortic valve disease (CAVD) is the most common heart valve disorder, yet its mechanism remains poorly understood. Valve interstitial cells (VICs) are the prevalent cells in aortic valve and their osteogenic differentiation may be responsible for calcific nodule formation in CAVD pathogenesis. Emerging evidence shows microRNA (miRNA, or miR) can function as important regulators of many pathological processes, including osteogenic differentiation. Here, we aimed to explore the function of miR-449c-5p in CAVD pathogenesis. In this study, we demonstrated the role of miR-449c-5p in VICs osteogenesis. MiRNA microarray assay and qRT-PCR results revealed miR-449c-5p was significantly down-regulated in calcified aortic valves compared with non-calcified valves. MiR-449c-5p overexpression inhibited VICs osteogenic differentiation *in vitro*, whereas down-regulation of miR-449c-5p enhanced the process. Target prediction analysis and dual-luciferase reporter assay confirmed Smad4 was a direct target of miR-449c-5p. Furthermore, knockdown of Smad4 inhibited VICs osteogenic differentiation, similar to the effect observed in up-regulation miR-449c-5p. In addition, animal experiments proved indirectly miR-449c-5p could alleviate aortic valve calcification. Our data suggested miR-449c-5p could function as a new inhibitory regulator of VICs osteogenic differentiation, which may act by targeting Smad4. MiR-449c-5p may be a potential therapeutic target for CAVD.

## Introduction

Calcific aortic valve disease (CAVD) is associated with significant cardiovascular morbidity and mortality in the elderly and results in severe end-stage cardiovascular dysfunction. To date, there are no effective drugs to prevent or treat CAVD^1, 2^. Valve replacement is the only available clinical option^3^. CAVD was previously recognized as a passive and degenerative consequence of aging; however, accumulating evidence has shown its pathogenesis is an active process involving many pathological changes^4–6^. The identification of its mechanisms can help treat CAVD.

Valve interstitial cells (VICs) play an important role in maintaining normal valve structure and function and are the predominant cell type in aortic valves^7^. Studies have shown VICs acquire an osteogenic phenotype during valve remodeling in CAVD and may be responsible for the formation of calcific nodules observed in the diseased valves^8, 9^. Prevention of VICs transformation may decrease calcification in CAVD. However, the specific pathways governing this process are poorly understood.

MicroRNA (miRNA, or miR) can act as fine-tuners in the regulation of diverse biological and pathological processes, including proliferation, apoptosis and differentiation, and miRNA dysregulation often results in impaired cellular function^10–13^. It has been reported some miRNAs are involved in smooth muscle cell calcification and myocardial fibrosis^14–16^. However, the function of miRNA in CAVD is still under investigation. Here, we aimed to identify a new regulator of CAVD and explore its function in disease progression. Our findings may provide clues for the pharmacological intervention for CAVD.

## Results

### Characteristics and phenotypes of VICs

**Figure 1:**
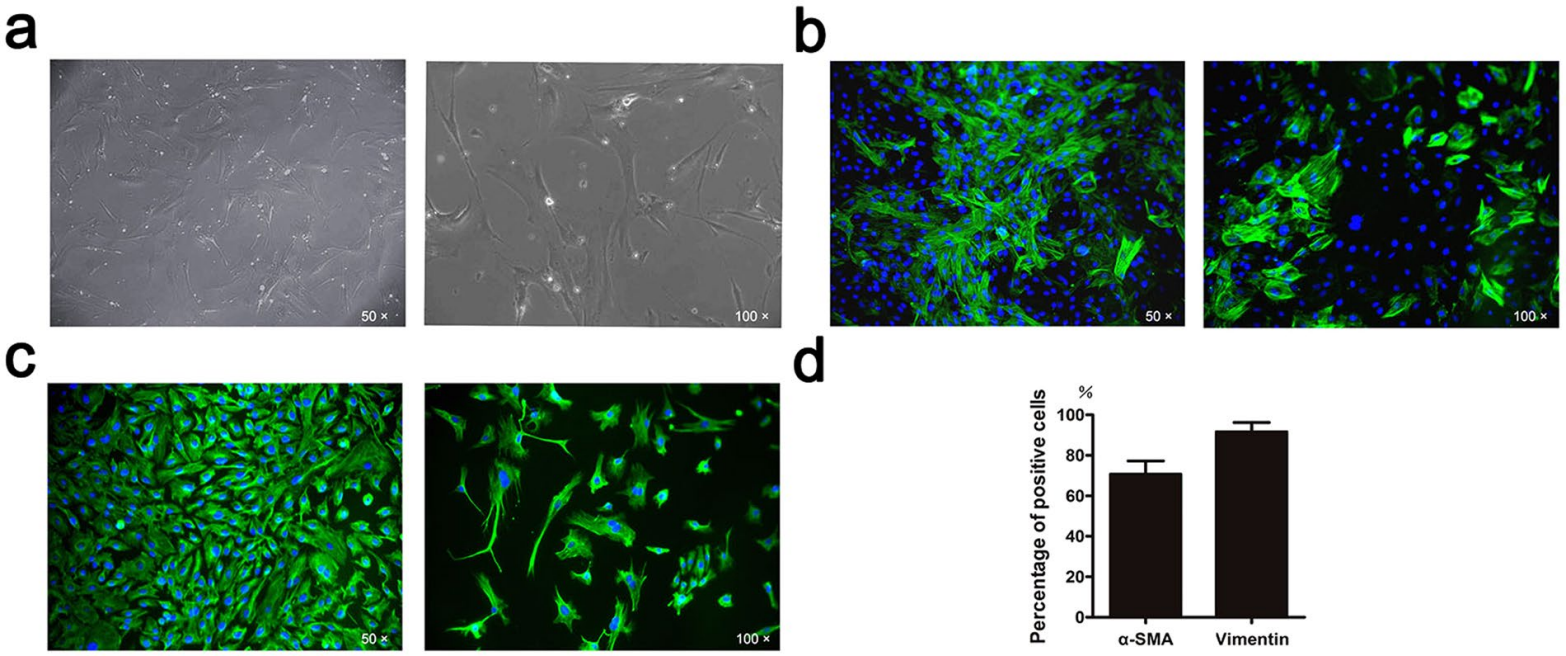
Characteristics and phenotypes of VICs. (**a**) The morphology of VICs (Left: low magnification; Right: high magnification). (**b**) Immunohistochemical staining of α-SMA (Left: low magnification; Right: high magnification). (**c**) Immunohistochemical staining of Vimentin (Left: low magnification; Right: high magnification). (**d**) Quantification of positive staining of α-SMA and Vimentin.

The primary cells began to grow with adherence at about 48 h of primary culture. After 6 days of primary culture, the adherent cells were flat and spindle-shaped. The cells grew slowly and reached to almost 70% confluences after 14 days of culture. During subsequent cell passages, the cell density was high and the cells were swirling and radial arranged (Fig. 1a).

To further confirm the isolated cells were VICs, two marker proteins associated with VICs were characterized by immunohistochemical staining. VICs from passage 3 were positive for α-SMA and vimentin (71% and 91%, respectively), showed in Fig. 1b,c,d.

### miR-449c-5p is down-regulated in human calcified aortic valves

**Figure 2:**
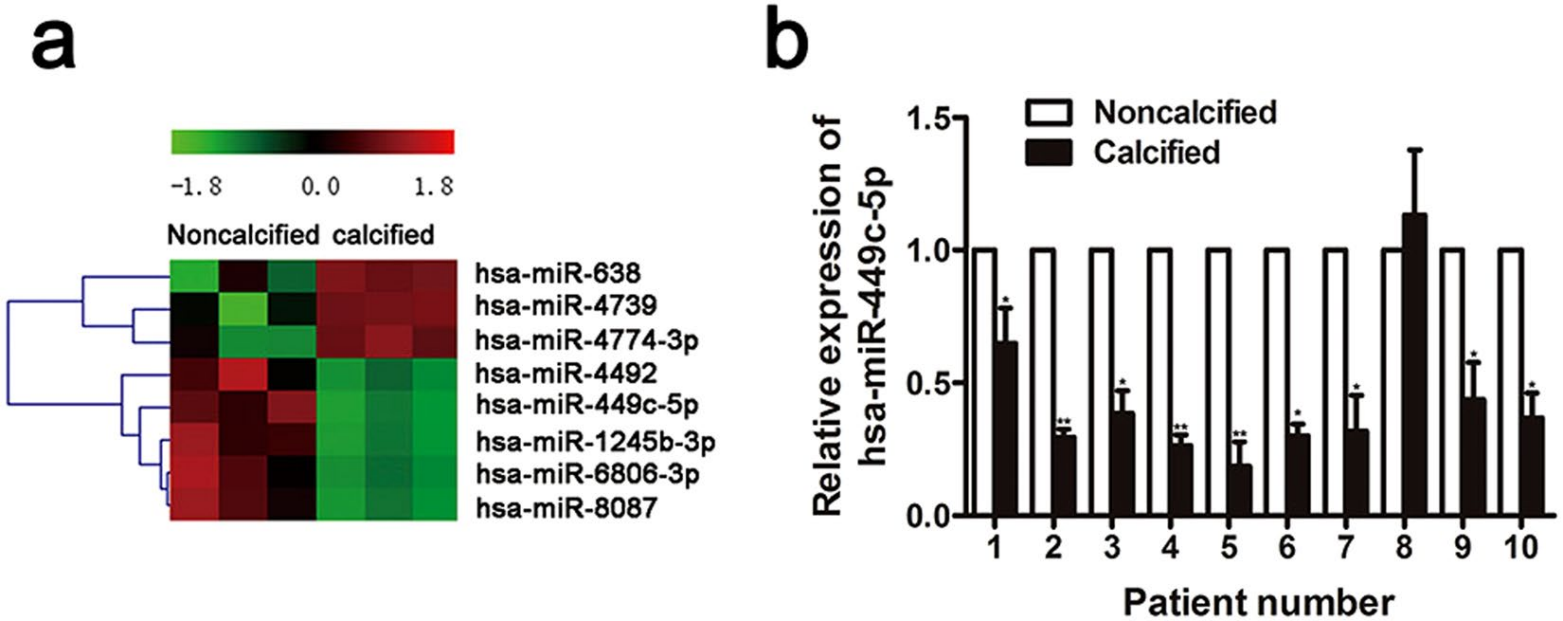
MiR-449c-5p is downregulated in human calcific aortic valves. (**a**) Heat map based on the differential expression of miRNAs between non-calcific and calcific aortic valves calculated by microarray. n = 3 in each group. (**b**) Verification of miR-449c-5p expression by qRT-PCR in non-calcific and calcific valves from 10 CAVD patients. Data are presented as the mean ± SD. *P < 0.05, **P < 0.01.

In order to identify the dysregulated miRNAs in CAVD pathogenesis, we analyzed the expression profile of miRNAs in non-calcified and calcified valves using microarray assay (Fig. 2a). Calcified valves significantly upregulated several miRNAs (miR-638, miR-4739, miR-4774-3p) and downregulated some miRNAs (miR-4492, miR-449c-5p, miR-1245b-3p, miR-6806-3p, miR-8087). Target gene prediction of these miRNAs was conducted using miRNA databases (TargetScan, PicTar, and miRanda). Interestingly, one of the predicted targets of miR-449c -5p is Smad4, which is a pivotal signaling molecule in the TGF-β/Smad pathway and mediates osteogenesis. Thus, miR-449c-5p was selected for further functional study in this research.

To verify the accuracy of microarray data, MiR-449c-5p expression was analyzed by qRT-PCR. We analyzed the expression of miR-449c-5p in the same set of 10 pairs of surgically resected calcified aortic valves and their adjacent non-calcified valves. Our results showed miR-449c-5p expression in calcified aortic valves were significantly down-regulated than those in adjacent non-calcified valves (Fig. 2b), which suggests miR-449c-5p might participate in the pathogenesis of aortic valve calcification.

### miR-449c-5p inhibits VICs osteogenic differentiation

**Figure 3:**
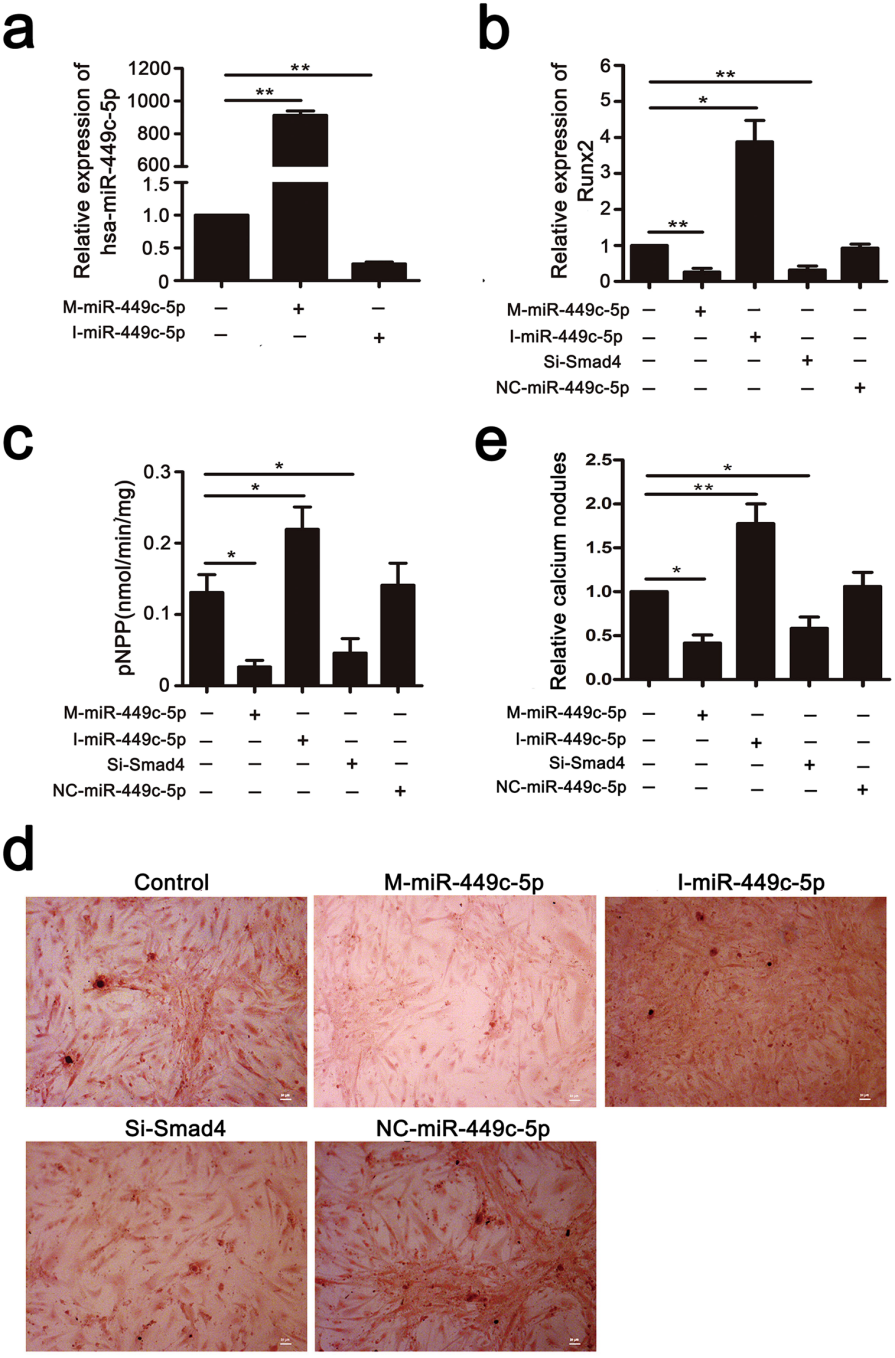
Overexpression of miR-449c-5p inhibits VICs osteogenic differentiation while downexpression of miR-449c-5p promotes the process. (**a**) qRT-PCR analysis of miR-449c-5p expression in VICs transfected with miR-449c-5p mimic or inhibitor at day 2. (**b**) qRT-PCR analysis of Runx2 expression at day 7 after osteogenic differentiation. (**c**) ALP activity at day 14 after osteogenic differentiation. (**d**) Alizarin red staining at day 14 after osteogenic differentiation. (**e**) Quantitative analysis of alizarin red staining. n = 3 in each group. Data are presented as the mean ± SD. *P < 0.05, **P < 0.01.

Firstly, we estimated the efficiency of miR-449c-5p transfection. Intracellular miR-449c-5p levels were significantly up-regulated by miR-449c-5p mimic and markedly down-regulated by miR-449c-5p inhibitor (Fig. 3a). To further investigate whether miR-449c-5p regulates VICs osteogenic differentiation, synthetic mimic and inhibitor of miR-449c-5p were transfected into VICs. Osteogenic capacity was examined by qRT-PCR, ALP activity and alizarin red staining. We detected the expression of Runt-related transcription factor 2 (Runx2) after transfection with miR-449c-5p mimic and inhibitor. Runx2 is a key transcription factor associated with osteogenic differentiation^17–19^. The Result showed that miR-449c-5p overexpression significantly suppressed Runx2 mRNA expression while miR-449c-5p inhibitor promoted the expression of Runx2 (Fig. 3b). ALP activity that are indicators of osteogenesis was also detected and miR-449c-5p overexpression significantly reduced ALP activity while miR-449c-5p low-expression increased ALP activity (Fig. 3c). In addition, alizarin red staining was used to analyze the calcium nodule formation. As shown in Fig. 3d,e, decreased calcium nodules were observed in miR-449c-5p overexpression group. These data demonstrated miR-449c-5p plays a role in inhibiting VICs osteogenic differentiation.

### miR-449c-5p directly targets Smad4

**Figure 4:**
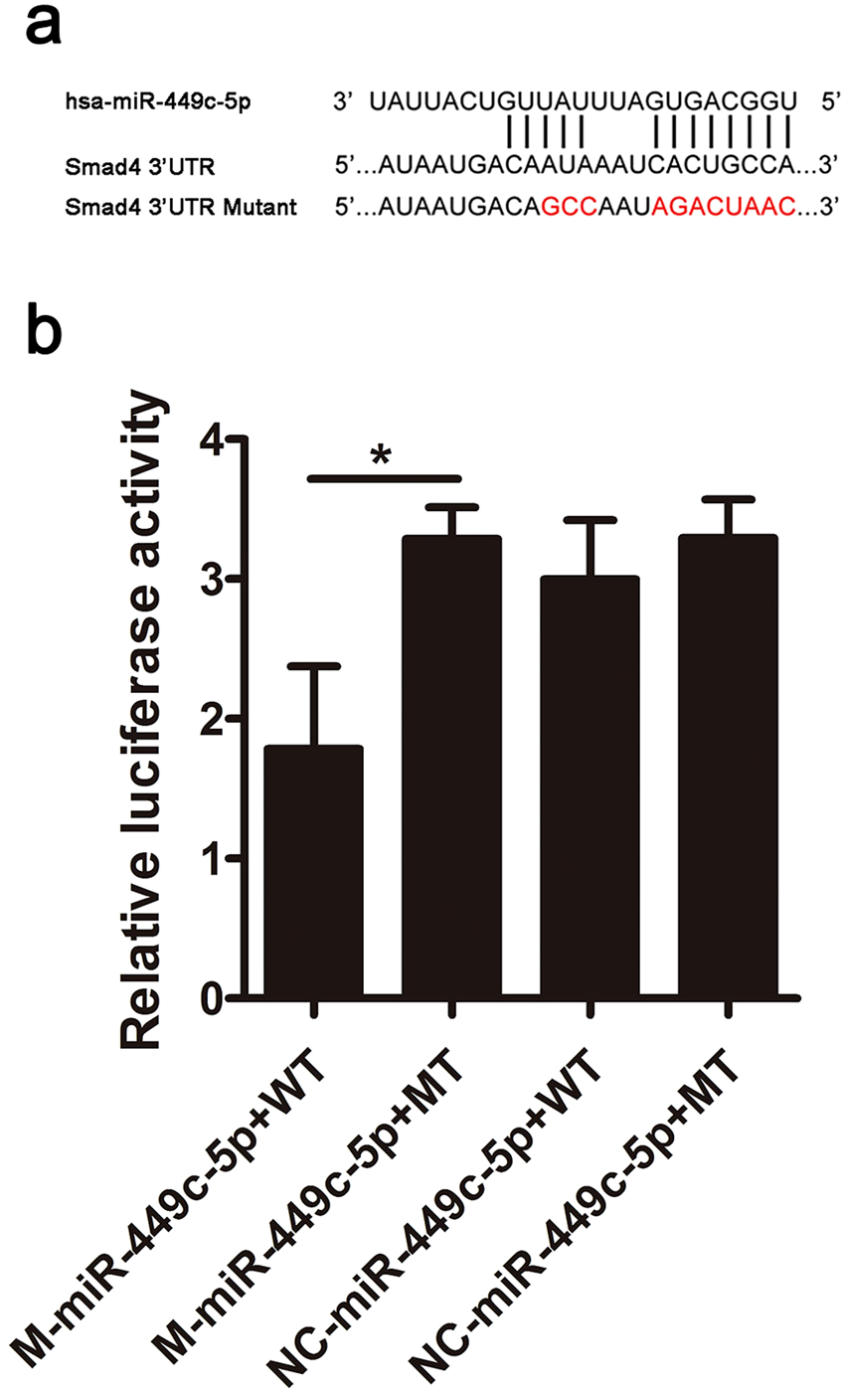
MiR-449c-5p directly targets Smad4. (**a**) Schematic of the putative miR-449c-5p target site in human Smad4 3′-UTR and the eleven mutated nucleotides are colored red. (**b**) Dual-luciferase assay after transfection of M-miR-449c-5p, NC-miR-449c-5p with wild-type Smad4 3′-UTR or mutant Smad4 3′UTR. n = 3 in each group. Data are presented as the mean ± SD. *P < 0.05.

**Figure 5:**
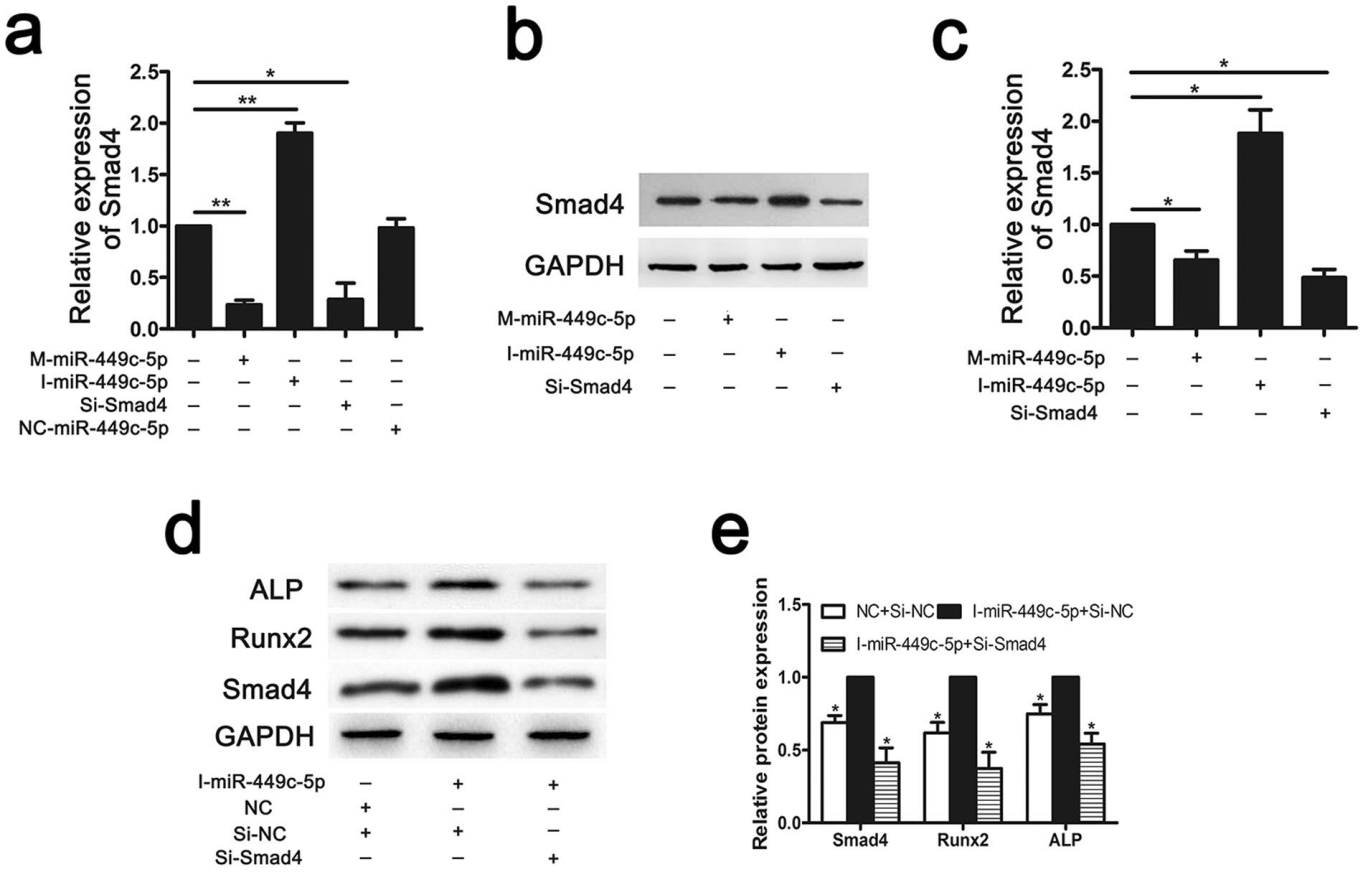
Regulation of Smad4 by miR-449c-5p during VICs osteogenic differentiation. (**a**) qRT-PCR analysis of Smad4 relative mRNA expression at day 7 after osteogenic differentiation. (**b**,**c**) Western blot analysis of Smad4 at day 14 after osteogenic differentiation. (**d**,**e**) Western blot further confirmed Smad4 knockdown could block the effect of miR-449c-5p inhibitor during VICs osteogenic differentiation. n = 3 in each group. Data are presented as the mean ± SD. *P < 0.05, **P < 0.01.

To reveal the molecular mechanism by which miR-449c-5p regulates osteogenic differentiation of VICs, TargetScan was used to predict potential targets of miR-449c-5p. Among the candidates, we found that Smad4 contains a miR-449c-5p binding sites in its 3′UTR (Fig. 4a). Given that, we firstly investigated whether miR-449c-5p mediated Smad4 expression during VICs osteogenic differentiation. Smad4 mRNA expression was significantly decreased by miR-449c-5p overexpression and markedly increased when the exprssion of miR-449c-5p was low after osteogenic differentiation 7 days (Fig. 5a). Furthermore, western blotting result showed Smad4 protein expression was significantly decreased due to miR-449c-5p overexpression and substantially increased by miR-449c-5p low-expression at day 14 after osteogenic differentiation (Fig. 5b,c). These data suggested miR-449c-5p attenuated Smad4 expression during VICs osteogenic differentiation.

To further determine whether miR-449c-5p directly targets Smad4, we constructed dual-luciferase reporters containing either wild-type (WT) or mutant (MT) Smad4 3′UTR (Fig. 4a). Our Results showed that miR-449c-5p mimic significantly repressed luciferase activity when cotransfected with reporter containing WT Smad4 3′UTR but not MT Smad4 3′UTR (Fig. 4b). These results indicate miR-449c-5p directly attenuated Smad4 expression through directly binding with Smad4 3′UTR.

### Smad4 downregulation inhibits osteogenic differentiation of VICs

To investigate the role of Smad4 on osteogenic differentiation of VICs, we suppressed Smad4 expression by transfecting VICs with SiRNA against Smad4 (Si-Smad4). As shown in Fig. 5a,b and c, both mRNA and protein expression of Smad4 were significantly reduced by Si-Smad4. Smad4 downregulation inhibits osteogenic differentiation of VICs, indicated by Runx2 mRNA expression (Fig. 3b), ALP activity (Fig. 3c) and alizarin red staining (Fig. 3d,e).

### Smad4 knockdown could block the effect of miR-449c-5p during VICs osteogenic differentiation

To confirm that the effect of miR-449c-5p during VICs osteogenic differentiation is mediated by targeting Smad4, we transfected miR-449c-5p inhibitor into VICs after Smad4 knockdown, and then proceeded with osteogenic differentiation. As is shown by western blotting (Fig. 5d,e), miR-449c-5p inhibitor could promote osteogenic differentiation in Si-Smad4 negative control (Si-NC) group, but differentiation in the presence of inhibitor is abolished after Smad4 knockdown. The result demonstrated that deletion of Smad4 could block the effect of miR-449c-5p inhibitor, further indicating that miR-449c-5p regulates VICs osteogenic differentiation through Smad4.

### miR-449c-5p inhibits aortic valve calcification *in vivo*

**Figure 6:**
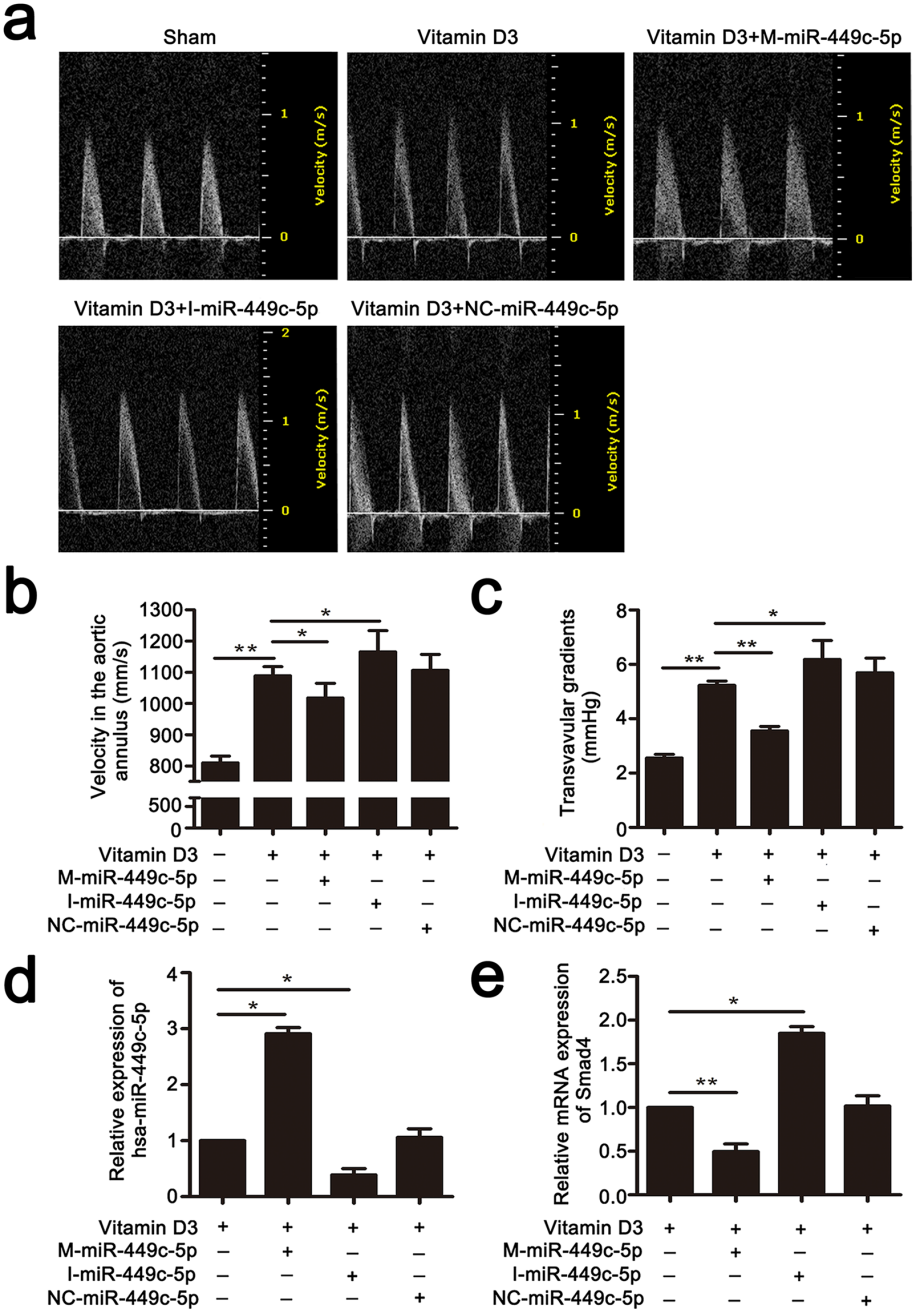
MiR-449c-5p inhibits aortic valve calcification *in vivo*. (**a**) The velocity profile in the aortic annulus measured by echocardiography six weeks after transfection in vitamin D3-treated mice. (**b**) Quantitative analysis of the velocity in the aortic annulus in each group. (**c**) Quantitative analysis of transvalvular gradients in each group. (**d**) qRT-PCR analysis of miR-449c-5p expression in the aortic valves of mice at day 5. (**e**) qRT-PCR analysis of Smad4 expression level in the aortic valves of mice at day 10. n = 7 in each group. Data are presented as the mean ± SD. *P < 0.05, **P < 0.01.

As miR-449c-5p inhibited VICs osteogenic differentiation, we finally investigated whether it could function in inhibiting aortic valve calcification *in vivo*. Firstly, we tested the miR-449c-5p expression of aortic valves at day 5 after tail intravenous injection. As shown in Fig. 6d, miR-449c-5p expression of aortic valves in mice could be significantly up-regulated by agomir-449c-5p and markedly down-regulated by antagomir-449c-5p. Next, we studied whether the change of miR-449c-5p expression in aortic valves of mice could influence Smad4 gene expression. Our results confirmed that Smad4 mRNA level was significantly decreased by miR-449c-5p overexpression and markedly increased by miR-449c-5p down-expression at day 10 (Fig. 6e). In the end, we explored the effect of Smad4 expression on the aortic valve calcification in mice by echocardiography. As shown in Fig. 6a,b,c, Vitamin D_3_-treated mice showed significantly higher velocity in the aortic annulus and higher transvalvular pressure gradients compared to sham mice, which indirectly confirmed aortic valve calcification was successfully induced. However, miR-449c-5p overexpression significantly reduced the velocity in the aortic annulus and the transvalvular pressure gradients compared to control group, while miR-449c-5p low-expression showed the opposite effect. In conclusion, these results demonstrated that miR-449c-5p could alleviate aortic valve calcification *in vivo*.

## Discussion

In this study, we for the first time identified the differentially expressed miRNAs in non-calcified and calcified aortic valves derived from 3 CAVD patients using miRNA microarray assay. We further investigated the function of the differentially expresses miRNA-449c-5p on the pathogenesis of CAVD *in vitro* and *in vivo*. MiR-449c-5p expression was markedly downregulated in calcified aortic valves. MiR-449c-5p overexpression inhibited VICs osteogenic differentiation, whereas the low-expression of miR-449c-5p enhanced its osteogenic potential *in vitro*. At last, the animal experiment was performed to further confirm the function of miR-449c-5p on aortic valve calcification *in vivo*. Thus, our study indicates that miR-449c-5p plays a critical role in VICs osteogenic differentiation by directly targeting Smad4.

MiRNAs are a wide family of evolutionarily conserved, small non-coding RNAs involved in post-transcriptional gene expression regulation^20^. Numerous studies have shown miRNAs could act as crucial modulators in cardiovascular diseases. Reinier *et al*. found age-induced miR-34a expression and inhibition of its target PNUTS is a key mechanism regulating cardiac contractile function during aging and after acute myocardial infarction^21^. Cui *et al*. identified miR-204 as a regulator of vascular smooth cell calcification *in vitro* and *in vivo* by targeting Runx2^14^. Another study validated miR-125b plays a central role in phenotypic switching and calcification of vascular smooth muscle cells by targeting Ets1^22^. Up to now, the central role of miRNAs in CAVD pathogenesis is getting more and more attention. For example, miR-141 blocked osteoblastic differentiation of porcine VICs through a BMP-dependent mechanism^23^. MiR-204 inhibits osteogenesis of human aortic VICs by directly targeting Runx2^24^. Zhang *et al*. confirmed a remarkable role for miR-30b in CAVD as a regulator of human aortic valve calcification and apoptosis^25^. In the present study, in order to explore the real miRNAs involved in CAVD pathogenesis, the miRNA microarray assay was first used to screen the differentially expressed miRNAs compared with the former studies. MiR-449c-5p was finally found and confirmed to negatively regulate the osteogenic differentiation of human aortic VICs.

The human miR-449 cluster is located on chromosome 5 in a highly conserved region within the second intron of the CDC20B gene. This cluster, which encodes the highly conserved miR-449, contains similar sequences and secondary structures as the miR-34 family, so they are classified as a single miRNA family^26^. Studies have confirmed miR-449 family members are primarily involved in tumor pathogenesis. One study confirmed miR-449a could inhibit proliferation and induce apoptosis by directly targeting E2F3 in gastric cancer^27^. Another study found miR-449b acts as a tumor suppressor in colon cancer stem cells through CCND1 and E2F3 down-regulation^28^. In recent years, it was reported in many studies that miR-449 family was closely related to osteogenic differentiation. MiR-34a and its target protein networks have been identified as critical modulator of osteogenic differentiation of human stromal stem cells. In addition, miR-34a was further proved to inhibit bone formation of human stromal stem cells *in vivo* ^29^. Previous studies have demonstrated that miR-34c is closely associated with osteogenic differentiation and bone development^30–32^. In this study, miR-449 family member miR-449c-5p was firstly identified to also function as a negative regulator in VICs osteogenic differentiation. The findings were consistent with previous studies about the function of miR-449 family on osteogenic differentiation. This study further expands our current knowledge of the importance of the miR-449 family in osteogenic differentiation and bone formation.

The cellular signals driving aortic valve calcification are reminiscent of those involved in osteogenic differentiation. Previous studies have indicated that the BMP-2/Smad signaling pathway is closely associated with osteogenic differentiation^33, 34^. Smad4 is the critical regulatory protein of the BMP-2/Smad signaling pathway. Smad4 could bind with phosphorylated Smad1/5/8 to form transcription complex and increase the expressions of downstream calcification gene targets^35^. More importantly, many studies have proved that Smad4 is an important regulator of osteogenic differentiation^36, 37^. In the present study, we have successfully demonstrated that Smad4 protein plays a crucial part in regulating VICs osteogenic differentiation. At the same time, we also proved that Smad4 was a direct target gene of miR-449c-5p using dual-luciferase reporter assay. A new study confirmed that LncRNA MALAT1 sponges miR-204 to promote osteoblast differentiation of human aortic VICs through up-regulating Smad4^38^. Our study also confirmed the function of Smad4 in regulating osteogenic differentiation of human aortic VICs.

Studies confirmed that calcified valves expressed numerous pro-inflammatory cytokines such as TNF, oxidized LDL cholesterol (oxLDL), and IL-1β/2/6^39, 40^. Husseini *et al*. verified IL-6 was overexpressed in stenotic aortic valves and VICs was an important source of IL-6^41^. Moreover, it was reported that during VICs mineralization, IL-6 was a key signal in promoting BMP2 production. In addition, inflammation is considered to be related to CAVD pathogenesis, and IL-6 has been shown as a proinflammatory factor to activate cardiovascular bio-mineralization and osteogenic signaling processes^6, 42–44^. Given all that, we preliminarily explored the relationship between IL-6 and miR-449c-5p expression. Our results showed secreted IL-6 progressively increased over time during VICs osteogenic differentiation (Supplementary Fig. 1a), suggesting that IL-6 may be involved in VICs osteogenic differentiation. In addition, we also confirmed miR-449c-5p expression significantly decreased over time after IL-6 stimulation (Supplementary Fig. 1b). Based on the above results, we hypothesized that IL-6 might participate in CAVD pathogenesis by interacting with miR-449c-5p expression. The underlying molecular mechanism needs to be further explored in our follow-up experiment.

In conclusion, we demonstrated that miR-449c-5p negatively regulated human aortic VICs osteogenic differentiation by directly targeting Smad4. This is the first report to study the regulatory role of miR-449c-5p for human aortic valve calcification *in vitro* and *in vivo*. Furthermore, our study suggested miR-449c-5p might be an effective candidate for the prevention and treatment of aortic valve calcification and provided an experimental basis for further noninvasive therapies to treat this disorder.

## Materials and Methods

### Ethics statements

The study protocol was approved by the Ethical Committee of Peking Union Medical College Hospital, and informed consents were obtained from the human donors. All experiments were performed in accordance with the relevant guidelines and regulations.

All animal experimental procedures were approved by the institutional review board of the Institute of Genetics and Developmental Biology, Chinese Academy of Sciences, and performed according to the Chinese Ministry of Public Health (CMPH) Guide for the care and use of laboratory animals.

### Calcific Aortic Valve Collection

Samples were obtained from 10 CAVD patients (Table 1), who had undergone aortic valve replacement. Exclusion criteria included non-stenotic, congenital aortic valve disease, autoimmune disease, genetic disease, and rheumatic aortic valve disease. Two samples were taken from each patient: one was calcified valves, and the other was non-calcified tissues that served as a control. All samples were resected during the operation and immediately placed in pairs in liquid nitrogen for the following study. At the same time, pathological examinations of tissue samples from 10 patients were performed to make sure the accuracy of tissues sampling and trimming.

**Table 1:** Demographic characteristics of the patients (n = 10).

Parameters	Value
Age (y, mean ± SD)	65.1 ± 4.86
Sex ratio (male/female)	5:5
Reason for aortic valve replacement (no.)	
Valve stenosis	7
Valve stenosis and insufficiency	3
Systemic disease (no.)	
Diabetes (Type 2)	3
Hypertension	2

### Microarray analysis

Calcified aortic valves and non-calcified valves from 3 CAVD patients were sent to carry on the miRNA microarray assay. Total RNA was extracted from tissues using the miRNAeasy Mini Kit (Qiagen GmbH). The miRNA microarray assay was performed by a service provider (LC Sciences). Total RNA (100 ng) was labeled with miRNA Complete Labeling and Hyb Kit (Agilent, USA) and hybridized on the Human miRNA Microarray Kit (Release 16.0, Agilent), which contains 60000 probes for 1205 and 144 human viral miRNAs. Hybridization signals were detected with the Agilent Microarray Scanner (Agilent, USA) and the scanned images were analyzed using Agilent Feature Extraction Software (Agilent, USA). Data were acquired by first subtracting the background noise of raw data from hybridization images and then normalizing using the LOWESS filter (locally weighted regression)^45^. Spot (standard deviation)/(signal intensity) < 0.5. Differentially expressed miRNAs were identified by a cutoff of fold change ≥ 1.5 and P < 0.01 by student’s *t*-test.

### MiRNA real-time quantitative PCR

MiR-449c-5p was extracted using the miRVana extraction kit (Ambion). For miR-449c-5p quantification, 10 ng total RNA was reverse transcribed and amplified using the miRNA reverse transcription and detection kit (Applied Biosystems, Inc.). All results were normalized to U6 levels, which were determined by the ABI miRNAU6 assay kit (Applied Biosystems, Inc.).

### VICs isolation and cell culture

Normal aortic valves (n = 5) were derived from patients who had undergone acute Stanford A aortic dissection. Primary aortic VICs were prepared as described previously^25, 46^. In brief, non-leaflet tissues were carefully eliminated after effective removal of the endothelial layer of aortic and ventricular aspects, then valves were immersed in 0.25% trypsin at 37 °C for 5 min. The tissues were then cut into pieces and digested for an additional 2 h at 37 °C. Primary VICs were obtained and seeded in growth medium (Dulbecco’s modified eagle medium supplemented with penicillin and streptomycin, mem non-essential amino acid, sodium pyruvate and 10% FBS) at 37 °C under a 5% carbon dioxide atmosphere. The purity of VICs was confirmed by microscopic examination and evaluation of the expression of marker proteins.

### Transient transfection and cell treatments

VICs were seeded at a density of 3 × 10^6^ cells in 6-well plates (Corning Costar, USA). When cells reached 70–80% confluence, VICs were individually transfected with 50-nmol/L miR-449c-5p mimic (M-miR-449c-5p), 50-nmol/L miR-449c-5p inhibitor (I-miR-449c-5p), 50-nmol/L miR-449c-5p negative control (NC-miR-449c-5p), or 50-nmol/L Smad4 siRNA (Si-Smad4) (GenePharma Co., Ltd, China) in OPTI-MEMI reduced serum medium (Invitrogen, USA) using lipofectamine 2000 (Invitrogen, USA) according to the manufacturer’s instructions. Transfection efficiency was measure in a preliminary test. Osteogenic differentiation was subsequently induced for 7 or 14 days after transfection by culturing cells in osteogenic differentiation medium (growth medium supplemented with 500-ng/ml BMP-2, 100-nmol/L dexamethasone, 50-μg/ml ascorbic acid, and 10-mmol/L β- glycerophosphate).

### mRNA quantitative real-time PCR

The mRNA expression of Runx2 and Smad4 were detected by using qRT-PCR after osteogenic induction 7 days. Total RNA was extracted with TRIzol reagent (Invitrogen). Power SYBR Green RT-PCR Kit (Invitrogen) and Bio-RAD CFX96 Real-Time System (Bio-RAD) were used for quantitative RT-PCR analysis. Data were normalized to the reference gene GAPDH for each cDNA sample. The primers used are listed in Table 2.

**Table 2:** Primers used in the research.

Gene name	Primer sequences
Smad4	Forward: GATACGTGGACCCTTCTGGA
	Reverse: CCTTTGCCTATGTGCAACCT
Runx2	Forward: GGACGAGGCAAGAGTTTCAC
	Reverse: TTCCCGAGGTCCATCTACTG
GAPDH	Forward: AGCCACATCGCTCAGACAC
	Reverse: TGGACTCCACGACGTACTC
U6	Forward: CTCGCTCGGCAGAACA
	Reverse: AACGCTTCACGAATTTGCGT
WT Smad4	Forward:TAGAGCTCTCCTGAGAGCTTGGTTGTTAATC
	Reverse: ACGTCTAGACAAGTATGGCTCTCCTTAAGC
MutantSmad4	Forward: TTGTTTAAACGAGCTCTCCTGAGAG
	Reverse: GTTAGTCTATTGGCTGTCATTATCTTTG
	Forward: GCCAATAGACTAACTATAACCTTGC
	Reverse: GACTCTAGACAAGTATGGCTCTCCTT

### Western blotting

The protein expression of Smad4, Runx2 and ALP were measured by using weatern blotting after osteogenic differentiation 14 days. The transfected VICs samples were fixed in 4% paraformaldehyde for 30 min, and then blocked with 0.2% Triton X-100 and 3% goat serum in PBS. Cell lysate was separated by 12% sodium dodecyl sulfate-polyacrylamide gelelectrophoresis (SDS-PAGE) gel. Primary antibodies included anti-Smad4 (ZSGB-BIO, China), anti-Runx2 (Cell Signaling Technology, USA), anti-ALP (Abcam, USA) and anti-GAPDH (ZSGB-BIO, China) were incubated overnight at 4 °C. After washing, membranes were incubated with secondary anti-rabbit or anti-mouse horseradish peroxidase-conjugated antibodies (ZSGB-BIO, China) for 2 h at room temperature.

### Dual luciferase reporter assay

The 3′-UTR of human gene Smad4 was amplified from human cDNA. The wide-type fragment containing the predicted miR-449c-5p binding site and its mutant fragment, designed to carry sites for SacI (5′end) and XbaI (3′end) at their ends, were obtained from 3′-UTR of Smad4. All primers are listed in Table 2. Amplicons were cleaved with SacI and XbaI and inserted between SacI and XbaI cleavage sites of pmirGLO vector (Promega, USA). 293 T cells were selected on the basis of the low endogenous miRNA expression. Cells were seeded in 24-well plates. When it reached to 70% to 80% confluence, the 800 ng wild-type or mutant reporter and 20-umol/L miR-449c-5p mimic, inhibitor or miR-NC (GenePharma Co., Ltd, China) were co-transfected into 293 T cells using Lipofectamine 2000 (Invitrogen, USA). 24 h after transfection, firefly and renilla luciferase activities were measured in cell lysates using the dual-luciferase reporter system (Promega, USA).

### Alkaline phosphatase activity assay

The osteogenic phenotype was determined based on the alkaline phosphatase (ALP) activity, which is an early osteoblastic differentiation marker. The ALP activity assay was conducted after osteogenic differentiation 7 days. Cells were washed twice with phosphate buffered saline solution (PBS) and lyzed with 150 μL NP-40 lysis buffer (Beyotime, China). The cell lysates were quantified by a alkaline phosphatase assay kit (Beyotime, China) using p-nitrophenyl phosphate (pNPP) as the substrate. In the presence of magnesium ions, pNPP was hydrolyzed by phosphatases to phosphate and p-nitrophenol. The rate of p-nitrophenol liberation is proportional to the ALP activity and can be measured photometrically. The ALP activity was measured by spectrophotometer at 405 nm.

### Alizarin red staining

Alizarin red staining was conducted after osteogenic differentiation 14 days to test matrix mineralization deposition, which appears at later stages of bone formation. In short, treated cells were washed twice with PBS, fixed in 95% ethanol for 10 min, washed with distilled water, and stained using Alizarin Red solution (1 g Tris and 0.1 g Alizarin Red (Sigma-Aldrich) in 100 ml ultrapure water; the pH was adjusted to 8.3 with HCl) at 37 °C for 30 min. Matrix calcification in alizarin red staining was manifested with red deposition.

### IL-6 ELISA assay

VICs were seeded at a density of 3 × 10^6^ cells in 6-well plates in growth medium. At 70–80% confluence, cells were induced with Osteogenic differentiation medium in experimental group. In control group, cells were only incubated in growth medium. Culture media were collected for quantitative detection of interleukin-6 (IL-6) on days 0, 1 and 2. IL-6 was measured using the human IL-6 platinum ELISA kit (eBioscience, USA) according to the manufacturer’s instructions.

### The influence of IL-6 on the expression of miRNA-449c-5p

VICs were plated at a cell density of 3 × 10^6^ cells in 6-well plates in growth medium. At 70%–80% confluence, cells were induced with growth medium containing 40-ng/ml IL-6 in experimental group. In control group, cells were only cultured in growth medium. Cellular RNA was extracted for miR-449c-5p detection on days 0, 1 and 2.

### Animal experiments

MiR-449c-5p mimic (agomir-449c-5p), inhibitor (antagomir-449c-5p) and negative control were purchased from GenePharma (GenePharma Co., Ltd, China). Thirty-five 10-week-old male Balb/c mice were randomly divided into five groups. Each group contained seven mice. Aortic valve calcification was induced by vitamin D_3_^47^. The mice were given subcutaneous injections of vitamin D_3_ at a dose of 500 000-IU/kg body weight to induce aortic valve calcification and received 5-mg/kg body weight of agomir-449c-5p, antagomir-449c-5p and negative control through tail intravenous injection at day 1–3. Saline was used as a control. The mice were killed on day 5 and aortic valves were obtained. The expression levels of miR-449c-5p in each group were detected by qRT-PCR. Besides, the expression of Smad4 in aortic valves was measured on day 10 by qRT-PCR. Six weeks later, velocity in the aortic annulus and transvalvular gradients were measured to indirectly evaluate aortic stenosis by using small animal echocardiography. The mice were anesthetized by intraperitoneal injection with 5% chloral hydrate, with the limbs and head fixed in the supine position, subsequently. The left ventricular images were collected horizontally at the aortic annulus of two-dimensional parasternal short-axis using an ultrasonic apparatus (Visualsonics, CA). Meanwhile, the M-mode echocardiogram was obtained under two-dimensional guidance, with over 10 cardiac cycles recorded. The echocardiogram was used to measure the velocity in the aortic annulus and transvalvular gradients.

### Statistical analysis

Each experiment was repeated in triplicate at least three times. Statistical analysis was performed by using SPSS 16.0. Data were presented as mean ± standard deviation (SD). Comparisons of parameters between two groups were evaluated by Student’s *t*-test. Comparisons of parameters among more than two groups were analyzed by one-way analysis of variance, and comparisons of different parameters between each group were made by a post hoc analysis using a Bonferroni’s test. Nonparametric Manne-Whitney U and Kruskal Wallis tests were performed when the sample size was smaller. Differences at *P* < 0.05 were considered to be statistically significant.

## Electronic supplementary material

Supplementary information
