# Supplementary material for: MicroRNA-449c-5p inhibits osteogenic differentiation of human VICs through Smad4-mediated pathway
Source: Sci Rep. 2017 Aug 18;7:8740. doi: 10.1038/s41598-017-09390-z (PMC5562804; doi:10.1038/s41598-017-09390-z)

MicroRNA-449c-5p inhibits osteogenic differentiation of human VICs through Smad4-mediated pathway

Rongjian Xu[1,a], Min Zhao[2,a], Yun Yang[3,4,a], Zhuo Huang[5], Chunying Shi[6], Xianglin Hou[3,7], Yannan Zhao[3,7], Bing Chen[3,7], Zhifeng Xiao[3,7], Jianzhou Liu[5], Qi Miao[5,*] and Jianwu Dai[3,7,*]

[1]*Department of Thoracic Surgery, The Affiliated Hospital of Qingdao University, Qingdao, China*

[2]*Center of Laboratory Medicine, Qilu Hospital of Shandong University (Qingdao), Qingdao, 266035, China*

[3]*State Key Laboratory of Molecular Developmental Biology, Institute of Genetics and Developmental Biology, Chinese Academy of Sciences, Beijing, China*

[4]*Graduate School, Chinese Academy of Sciences, Beijing, China*

[5]*Department of Cardiac Surgery, Peking Union Medical College Hospital, Peking Union Medical College, Chinese Academy of Medical Sciences, Beijing, China*

[6]*Institute for Translational Medicine, College of Medicine, Qingdao University, Qingdao, China*

[7]*Institute of Combined Injury, State Key Laboratory of Trauma, Burns and Combined Injury, College of Preventive Medicine, Third Military Medical University, 30, Gaotanyan Road, Chongqing, China*

[*]Corresponding authors: Dr.Jianwu Dai, Institute of Genetics and Developmental Biology, Chinese Academy of Sciences, 3 Nanyitiao, Zhongguancun, Beijing 100190, China, Tel: +86-10-82614426, Fax: +86-10-82614420, E-mail: jwdai@genetics.ac.cn; Dr. Qi Miao, Department of Cardiac Surgery, Peking Union Medical College Hospital, Peking Union Medical College, Chinese Academy of Medical Sciences, 1Shuaifuyuan,Wangfujing Street, Beijing100730, China, Tel: +86-10-69152820, Fax:+86-10-69156829,E-mail: miaoqipumc@hotmail.com

[a]These authors contributed equally to this work.

Supplementary Figure legends

Supplementary Figure 1. IL-6 may participate in regulating miR-449c-5p-associated VICs osteogenic differentiation.

(a) ELISA assay of IL-6 secreted by VICs after osteogenic induction. (b) qRT-PCR analysis of miR-449c-5p expression in VICs after IL-6 stimulation. n=3 in each group. Data are presented as the mean ± SD. **P*< 0.05. ***P*< 0.01.

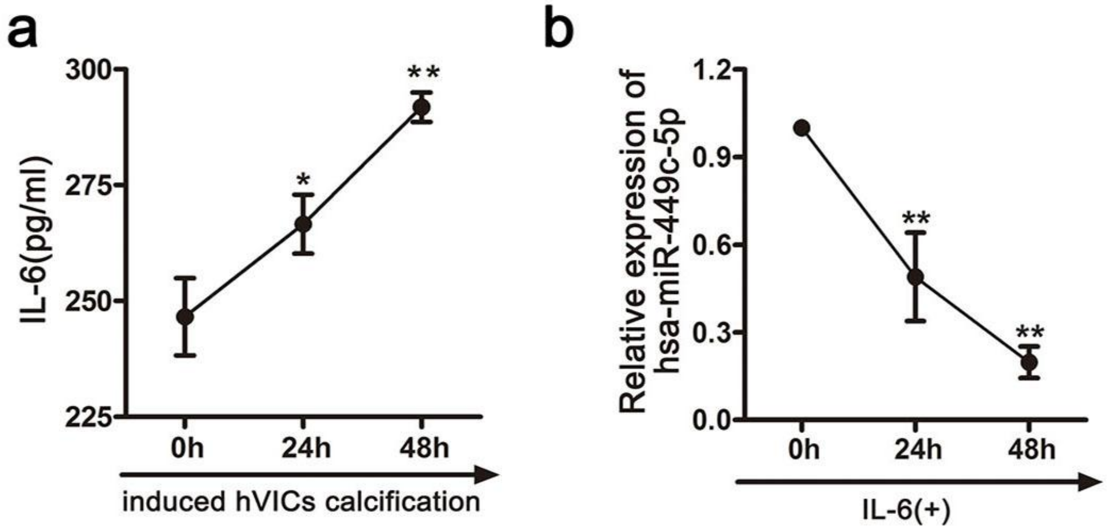

Supplement: Supplementary file 1 — Supplementary information [file 41598_2017_9390_MOESM1_ESM.pdf]
